# Predictors of poor glycemic control among patients with type 2 diabetes on follow-up care at a tertiary healthcare setting in Ethiopia

**DOI:** 10.1186/s13104-019-4248-6

**Published:** 2019-04-04

**Authors:** Gebre Teklemariam Demoz, Alem Gebremariam, Helen Yifter, Minyahil Alebachew, Yirga Legesse Niriayo, Gebremicheal Gebreslassie, Gebremariam Woldu, Degena Bahrey, Workineh Shibeshi

**Affiliations:** 1 School of Pharmacy, Aksum University, Po.Box: 298, Aksum, Ethiopia; 2 School of Public Health, Adigrat University, Adigrat, Ethiopia; 3 School of Medicine, Addis Ababa University, Addis Ababa, Ethiopia; 4 School of Pharmacy, Addis Ababa University, Addis Ababa, Ethiopia; 5 School of Pharmacy, Mekelle University, Mekelle, Ethiopia; 6 School of Nursing, Aksum University, Aksum, Ethiopia

**Keywords:** Type 2 diabetes, Fasting blood glucose, Glycemic control, Predictors, Ethiopia

## Abstract

**Objective:**

Contemporary clinical guidelines endorsed that glycemic control is the ultimate goal in the management patients with diabetes. The aim of this study was to assess the prevalence of glycemic control and to identify predictors of poor glycemic control in patients with type 2 diabetes (T2D). A cross-sectional study was conducted among systematically selected 357 diabetic patients. Data were collected through direct patients’ interviews and medical chart review. Binary logistic regression analyses were performed and analyzed using SPSS version 22.0.

**Results:**

Participants’ mean age was (± SD) 56.1 ± 11.6 years. Nearly four in five (77.9%) of the participants had comorbidities, mainly of hypertension, and 60.2% had diabetic complications, mainly diabetes neuropathy. Poor glycemic control was found in 68.3% of the participants with a mean (± SD) FBG of 174.1 ± 48.9 mg/dL. Being female gender, having greater body mass index and low medication adherence was significantly associated with poor glycemic control. In conclusion, the overall aspects of glycemic control level of patients were far from the standards. Being female, greater body mass index and poor medication adherence were predictors of poor glycemic control. In response to this finding, an aggressive intervention that targets in improving the glycemic control is required.

**Electronic supplementary material:**

The online version of this article (10.1186/s13104-019-4248-6) contains supplementary material, which is available to authorized users.

## Introduction

Diabetes is a chronic metabolic disorder characterized by persistent hyperglycemia due to a deficiency in insulin secretion, insulin action or both [1–3]. Diabetes is one of the leading health problems of this century. The prevalence of type 2 diabetes (T2D) is increasing over time. Likewise, the prevalence of people living with diabetes in Ethiopia is substantially increased from time to time. Thus, increased from 3.8% in 2014 to 5.2% in 2017 [3–5].

Glycemic control is the ultimate goal of management of diabetes [2, 6]. Adequate glycemic control helps to reduce or delay the burden of diabetes complications [7]. According to the International Diabetes Federation and the American Diabetes Association guidelines, glycated hemoglobin (HgA1c) value is the most recommended monitoring parameter for appropriate glycemic control status. Thus, the value of HgA1c within the last 3 months is indicators of patients’ glycemic control [2, 8]. Many studies reveal an association between HgA1c values and diabetes complications. Reducing HgA1c values significantly decreases diabetes complications and the overall death from diabetes [9–11]. Thus, early and adequate glycemic control improves macrovascular outcomes [12] and diabetes complications [13–15].

Achieving optimal glycemic level may not be an easy task. It depends on the type of treatment received patients’ adherence and comorbidities [2, 3]. Likewise, risk factors, obesity, biological and psychosocial factors are responsible for differences in glycemic control [16]. Limited data are currently available that evaluate the relationship of glycemic control with lifestyle, clinical characteristics, and treatment pattern. Hence, the primary purpose of this study was to identify predictors of glycemic control status in T2D patients.

## Main text

### Methods

#### Study design and study setting

A hospital-based cross-sectional study design was conducted from August 2017 through July 2018 at Tikur Anbessa Specialized Hospital (TASH). This hospital is the largest public referral and teaching hospital in Ethiopia which is affiliated to Addis Ababa University. The hospital has about 800 beds and is in the provision of complicated diagnostic and treatment services for about 500,000 patients a year.

#### Study population

**Fig. 1:**
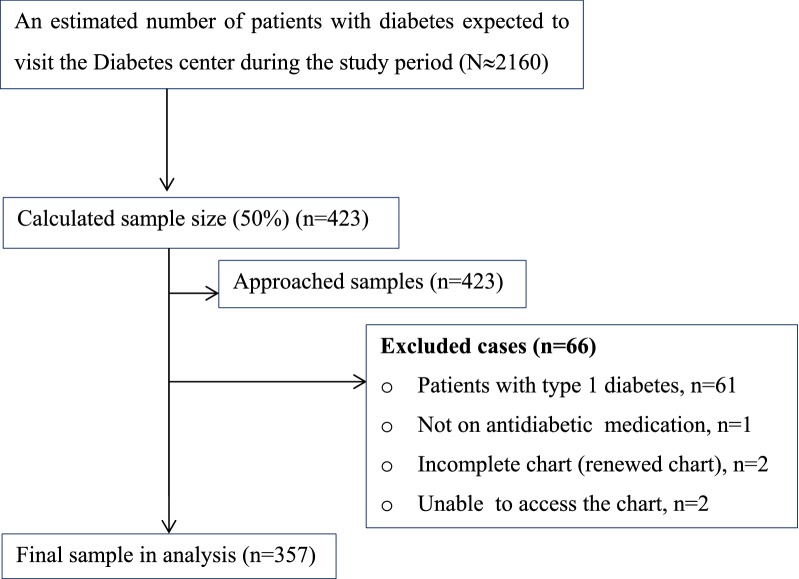
Summary of study participants recruiting flow chart

During the 3 months’ study period, about 2160 diabetes patients were expected to visit the diabetes clinic (Fig. 1). We calculated the minimum sample size of participants using a single population formula, by taking 50% of population and found 384, adding 10% contingency = 423. Out of these, 357 patients were selected using systematic sampling technique. The inclusion criteria were confirmed cases of T2D aged 18 years and above, ambulatory patients with T2D who were taking at least one antidiabetic drug, patients with T2D who had regular follow-up in the diabetes clinic for at least 1 year. Data were collected through patient interview using structured questionnaire designed for this study. The questionnaire was included questions about sociodemographic details, (includes as gender, age, marital status, level of education and employment status), questions about the patients’ clinical characteristics (includes diabetes complications, comorbidities and duration of diabetes) and questions about the patients’ lifestyle details (includes physical activities, alcohol use, smoking and dietary plan). Variables extracted from medical chart were medications taken by the patients for diabetes and other comorbidities, laboratory values for diabetes and related medical conditions. Anthropometric measurement values; includes height and weight, body mass index (BMI) and waist circumference (WC) were obtained at the time of interview.

#### Data processing and analysis

Data were entered and analyzed using EpiData Manager Version 4.0.2.00 (EpiData Association, Denmark) [17] and SPSS version 22.0 (SSPS Inc., Chicago, Illinois, USA), respectively. Descriptive statistics and binary logistic regression model was used to investigate predictors of poor glycemic control and presented using Odds Ratios (ORs) with its 95% confidence intervals (CIs). Statistical significance was considered at p-value < 0.05.

#### Definitions

*Adequate glycemic control* it was defined as average fasting blood glucose measurement between 70 and 130 mg/dL or HbA_1c_ < 7%.

*Poor glycemic control* it was defined as patients whose average blood glucose measurements of the three consecutive visits was above 130 or below 70 mg/dL or HbA_1c_ > 7% [2, 3, 8].

### Results

#### Sociodemographic and clinical characteristics

A total of 357 study participants were included in the study. The mean (± SD) age of the study participants was 56 ± 11 years. More than half, 189 (52.9%) of the participants were females (Table 1). The clinical characteristics of patients with T2D are described in Table 1. The mean duration of the diabetes disease since diagnosis was 11.64 ± 6.95 years. Comorbidity in these study participants was considerable, 278 (77.9%). The most common comorbidity was hypertension, 188 (65.5%), followed by dyslipidemia 171 (60.9%) and ischemic heart disease 37 (13.1%). Moreover, 215 (60.2%) participants had developed at least one chronic diabetes complications. Furthermore, more participants (60.2%) with poor glycemic control had one or more diabetes complication. Diabetic neuropathy was the most (46.8%) commonly reported diabetes complications.

**Table 1:** Sociodemographic and clinical characteristics of T2D patients on follow-up at diabetes center, Ethiopia, 2018

Category	Subcategory	Glycemic control		Total (%)	p-value
		Poor	Good		
Sex	Male	74 (30.2)	94 (83.2)	168 (47.1)	
	Female	170 (69.8)	19 (16.8)	189 (52.9)	0.012
Age group	18–40	14 (5.7)	8 (7.1)	22 (6.2)	0.12
	41–60	134 (54.9)	54 (47.8)	188 (52.7)	
	> 60	96 (39.3)	51 (45.1)	147 (41.7)	
Marital status	Never married	8 (3.3)	5 (4.4)	13 (3.6)	0.59
	Ever married	236 (96.7)	108 (95.6)	344 (96.4)	
Residence	Urban	206 (84.4)	98 (86.7)	304 (85.2)	0.57
	Rural	38 (15.6)	15 (13.3)	53 (14.8)	
Education	No formal education	33 (13.5)	17 (15.0)	50 (14.0)	0.52
	Primary (1–8)	47 (19.3)	16 (14.2)	63 (17.6)	
	Secondary (9–12)	66 (27.0)	37 (32.7)	103 (28.9)	
	Tertiary (graduates)	98 (40.2)	43 (38.1)	141 (39.5)	
Employment	Employed	135 (55.3)	66 (58.4)	201 (56.3)	0.59
	Unemployed	109 (44.7)	47 (41.6)	156 (43.7)	
Duration of diabetes	Mean (± SD)	13.01 ± 3.11	10.24 ± 2.13	11.64 ± 6.95	0.051
Presence of comorbidities	Yes	195 (79.9)	83 (73.5)	278 (77.9)	0.115
	No	49 (20.1)	30 (26.5)	79 (22.1)	
Types of co morbidities	Hypertension	130 (53.3)	58 (51.3)	188 (65.5)	0.381
	Dyslipidemia	123 (50.4)	48 (42.5)	171 (60.9)	0.222
	IHD	27 (11.1)	10 (8.8)	37 (13.1)	0.414
	Others^a^	32 (13.1)	18 (15.9)	50 (17.7)	0.201
Diabetes complications	Yes	193 (79.1)	22 (19.5)	215 (60.2)	0.018
	No	51 (20.9)	91 (80.5)	142 (39.8)	
Types of complications	Neuropathy	143 (58.6)	24 (21.2)	167 (46.8)	0.036
	Nephropathy	62 (25.4)	6 (5.3)	68 (19.1)	0.374
	Retinopathy	45 (18.4)	5 (4.4)	50 (14.0)	0.262
FBG, mg/dL	Mean (± SD)	186.8 ± 47.4	146.6 ± 39.9	174.10 ± 48.9	< 0.001
BMI (kg/m^2^)	Mean (± SD)	29.4 ± 6.1	24.9 ± 4.5	27.15 ± 4.6	0.217

^a^Thyroid disorders, peptic ulcer disease, asthmatic

Moreover, the mean (± SD) BMI of participants was 27.15 ± 4.6 kg/m^2^. Of these, the majority (76%) of the obese (> 30 kg/m^2^) participants were females. However, more than 70% of the participants have been reported as they are adherent to regular physical activity.

#### Prescribed medication profiles

More than half (53.8%) of participants were on oral glucose-lowering drugs (OGLD) alone (with no insulin as a component of their drug therapy), followed by OGLD plus insulin (27.7%) and insulin alone, with no OGLD (18.5%). The most frequently prescribed antidiabetic agent was metformin (78.4%) (Additional file 1). In addition, glycated hemoglobin (HgA1c) is the gold standard for monitoring blood sugar, but in our clinic very few (14.8%) of the participants had HgA1c results. The mean of FBG level for the last three consecutive visits was 174.1 ± 48.9 mg/dL. Of the studied participants, 68.3% of participants were found to have poor glycemic control.

#### Predictors of poor glycemic control

As illustrated in Table 2, the predictors statistically significant with poor glycemic control in the multivariable analysis were being female gender, BMI (> 30 kg/m^2^) and poor medication adherence. From the AOR, being female gender (AOR = 1.59, 95% CI 1.20–2.38, p = 0.041) was positively associated to have poor glycemic control. Moreover, the odds of poor glycemic control among participants who had obese was increased by more than three times (AOR = 3.51, 95% CI 1.82–4.01, p = 0.027) compared to participants with normal body weight. On the other hand, the odds of poor glycemic control among patients who had poor adherence to their medication were five times (AOR = 5.10, 95% CI 1.18–6.55, p = 0.001) more than patients who had high adherence to their medication.

**Table 2:** Predictors for poor glycemic control in patients with T2D on follow up at diabetes center, Ethiopia, 2018

Category	Subcategory	Glycemic control level		COR (95% of CI)	AOR (95% of CI)	p-value
		Poor	Good			
Sex	Male	74 (44.0)	94 (56.0)	1	1	
	Female	170 (89.9)	19 (10.1)	1.25 (1.8–2.21)	1.59 (1.20–2.38)	0.041**
Age	Mean (± SD)	56 ± 11	57 ± 12	2.11 (0.81–1.75)	1.57 (1.11–2.31)	0.146
Marital status	Never married	8 (61.5)	5 (38.5)	0.89 (0.55–1.47)	0.93 (0.81–1.35)	0.593
	Ever married	236 (68.6)	108 (31.4)	1	1	
Education	No formal education	33 (66.0)	17 (34.0)	2.81 (0.21–0.89)*	1.59 (0.37–1.09)	0.061
	Primary (1–8)	47 (74.6)	16 (25.4)	2.51 (0.31–1.53)	2.10 (0.75–1.77)	0.089
	Secondary (9–12)	66 (64.1)	37 (35.9)	1.22 (0.51–1.28)	1.11 (0.55–1.31)	0.102
	Tertiary (graduates)	98 (69.5)	43 (30.5)	1	1	
Exercise	No	81 (77.1)	24 (22.9)	3.71 (0.24–0.87)*	2.92 (0.78–1.10)	0.092
	Yes	163 (64.7)	89 (35.3)	1	1	
Complications	No	51 (35.9)	91 (64.1)	1	1	
	Yes	193 (89.9)	22 (10.1)	2.20 (0.44–.88)*	2.0 (0.69–1.06)	0.074
BMI (kg/m^2^)	Normal	15 (45.5)	18 (54.5)	1	1	
	Overweight	94 (64.8)	51 (35.2)	1.49 (0.23–0.91)*	1.68 (1.01–2.55)	0.061
	Obese	135 (75.4)	44 (24.6)	2.88 (1.27–2.86)	3.51 (1.82–4.01)	0.027**
Lipid control	Good	45 (35.4)	82 (64.6)	1	1	
	Poor	199 (86.5)	31 (13.5)	2.35 (0.29–0.83)*	2.13 (0.57–1.34)	0.088
BP control	Controlled	43 (35.5)	78 (64.5)	1	1	
	Uncontrolled	201 (85.2)	35 (14.8)	3.41 (0.61–1.33)	4.51 (0.89–1.94)	0.112
Antidiabetics	Oral alone	13 (1.4)	55 (28.6)	1	1	
	Oral + insulin	71 (71.7)	28 (28.3)	1.11 (0.29–0.97)*	1.81 (0.59–1.64)	0.067
	Insulin	36 (54.5)	30 (45.5)	0.85 (0.66–1.20)	0.96 (0.79–1.38)	0.091
Adherence	Low adherence	102 (65.0)	55 (35.0)	4.59 (1.13–4.58)	5.10 (1.18–6.55)	0.001*
	High adherence	65 (73.0)	24 (27.0)	1	1	
Dietary plan	Poor adherence	192 (91.0)	19 (9.0)	2.91 (0.31–0.85)*	3.44 (0.71–1.55)	0.098
	Good adherence	62 (39.7)	94 (60.3)	1	1	

*COR* crude odds ratio, *AOR* adjusted odds ratio, *SD* standard deviation, *BMI* body mass index, *BP* blood pressure

* Indicates p ≤ 0.05 in the univariate and ** ≤ 0.05 in the multivariate analysis

### Discussion

From this study, we found that more than two-thirds (68.9%) of the patients had poor glycemic control. This proportion is comparable with studies conducted (70.9%) by Kassahun et al. [18] and 71.9% by Hailu et al. [19]. However, this finding was lower than that found from other Ethiopian studies that reported as 81.7% by Angamo et al. [20]. In addition, this percentage was higher than the percentages found in Ethiopian (61.5%) [21] and Jordanian (60.8%) patients with T2D [10]. This level of clinical outcome may require a comprehensive approach to working with patients to achieve the intended glycemic level.

Furthermore, more than half (53.8%) of participants were on oral OGLD alone (with no insulin as a component of their drug therapy). This percentage was consistent with the finding (54.4%) reported by Weng et al. [22]. The most frequently prescribed antidiabetic agent used as monotherapy and/or combination therapy with at least one other oral and/or insulin was metformin (78.4%) [23]. Thus, it seems to suggest that some level of clinical inertia, where physicians might be slow in responding to the clinical parameters. Indeed, a lack of achieving glycemic control might also be explained by missing of the right drug therapy, although 26.1% of participants were receiving two or more antidiabetic agents in their recent visit. This allows in evaluating the effect of different characteristics and medications and to make the necessary regimen change and dose adjustment to achieve the anticipated goals of therapy.

Results obtained from multivariable analysis indicated that differences between participants due to factors associated with poor glycemic control were being female, greater BMI (> 30 kg/m^2^) and low to medium medication adherence. Moreover, females with T2D were more likely to have poor glycemic control. This was consistent with the study conducted in Ethiopia [21] and abroad [24]. In fact, in our study, a low dose of antidiabetics that need to be titrated their dose was higher in female participants than male participants (67.2% versus 23.8%, p = 0.03). This is agreed with the study found that women had needed a higher insulin dose/kg than men [25]. Hence, we recommend that prescribers need to be aware of the need to titrate insulin dosing and intensify the treatment pattern, principally in women participants with poor glycemic control as they are more obese and accumulated bad cholesterol than men with diabetes, to achieve the intended glycemic target. In the present study, the reason could be due to: (1) Ethiopian female might not attend their follow up therapy as needed as male due to additional workload in home and thus be less likely to follow their drug therapy attentively (2) less adherent to their lifestyle modification therapies, like regular physical activity. In fact, this could be related with obesity, 76% of participants with > 30 kg/m^2^ BMI was found among female participants of this study. A good explanation for such issues was supported by WHO [4] and Lester [26], which reported more physical inactivity, obesity and overweight were observed in Ethiopian females than males. This is in agreement with three different studies, implies fewer women were achieved their glycemic target compared to men [25, 27, 28]. Gender difference influences the liability to diabetes therapies; negatively affect accessing health services and amplify the impact of diabetes on them. Thus, many women in the world had poor accesses to treatment, care and education [29]. Lifestyle modification is an important non-pharmacological therapy in reducing risks for patients with diabetes. In this study, we found that the odds of participants with poor glycemic control were also increased with BMI (≥ 30 kg/m^2^). In line with studies reported in Tehran as waist circumference was a predictor for poor glycemic control in female patients with T2D [30] and the percentage of patients with poor glycemic control increased as BMI increases [22].

In line with a study carried out by Kassahun et al. [18], in our study, poor medication adherence was also found as an important predictor of poor glycemic control.. This could be an indicator, in which patients might have poor knowledge about their illness, medications, and/or poor provision of counseling service. These outlooks are questionable, whether health care providers are not doing what was needed to support their patients in creating awareness their illness and prescribed medications or patients’ related factors. This study was highlighted that poor adherence is an important factor for poor glycemic control. However, medication adherence should not be supposed as the patient’s problem alone. It might due to frustration to agree on the prescription entirely with the patient and/or failure to provide continuous support that the patient needs once the drug has been dispensed. Hence, need to establish patients’ perspective in ensuring and inspiring to discuss the aim of their drug therapy to resolve such problems related to medication adherence.

### Conclusions

More than two-thirds of the study participants had poor glycemic control which is far below the recommended standards. Being female gender, obese (BMI > 30 kg/m^2^) and poor medication adherence was predictors for poor glycemic control. Thus, seems to emphasize the need for a modification in the approach and strategies in diabetes care in achieving the intended glycemic target.

## Limitations of the study

The inherent nature of cross-sectional retrospective study design that does not show a temporal association could be a limitation. Another limitation was FBG measurements; obtained from medical records that may be subjected to measurement errors could be lead to underestimated or overestimated. However, effort was made to overcome this issue by taking the mean average of the last three consecutive visits of FBG measurements.

## Additional file

**Additional file 1.** Medication profiles among patients with T2D on follow up at diabetes center, Ethiopia, 2018.

## Abbreviations

ADA: American Diabetes Association
ADEs: adverse drug events
CVD: cardio vascular disease
eGFR: estimated glomerular filtration rate
FBG: fasting blood glucose
IDF: International Diabetes Federation
LLAs: lipid-lowering agents
OGLD: oral glucose-lowering drugs
SMBG: self-monitoring of blood glucose
TASH: Tikur Anbessa Specialized Hospital
WHO: World Health Organization
