# Supplementary material for: Predictors of poor glycemic control among patients with type 2 diabetes on follow-up care at a tertiary healthcare setting in Ethiopia
Source: BMC Res Notes. 2019 Apr 4;12:207. doi: 10.1186/s13104-019-4248-6 (PMC6449968; doi:10.1186/s13104-019-4248-6)
Supplement: Supplementary file 1 — Additional file 1. Medication profiles among patients with T2D on follow up at diabetes center, Ethiopia, 2018. [file 13104_2019_4248_MOESM1_ESM.docx]

| Medication profiles among patients with T2D on follow up at diabetes center, Ethiopia, 2018 | | | | | |
| --- | --- | --- | --- | --- | --- |
| Category | Subcategory | Status of glycemic control | | Total (%) | P-value |
|  |  | Poor, n =244 | Adequate, n=113 |  |  |
| Antidiabetics | OGLD alone | 137(56.1) | 55(48.7) | 192(53.8) | 0.028 |
|  | Metformin alone | 98(40.2) | 31(27.5) | 129(36.1) |  |
|  | Metformin + Glibenclamide | 31(12.6) | 20(17.7) | 51(14.3) |  |
|  | Metformin +Glimepiride | 8(3.3) | 4(3.5) | 12(3.4) |  |
|  | Insulin alone | 36(14.8) | 30(26.5) | 66(18.5) | 0.512 |
|  | OGLD + Insulin | 71(29.1) | 28(24.8) | 99(27.7) | 0.251 |
|  | Metformin + Insulin | 67(27.5) | 21(18.6) | 88(24.6) |  |
|  | Dapagliflozine + insulin | 2(0.08) | 7(6.2) | 9(2.5) |  |
|  | Glibenclamide + insulin | 2(0.08) | 0(0.0) | 2(0.06) |  |
| Angiotension converting enzyme inhibitors | | 119(48.8) | 47(41.6) | 166(46.5) | 0.206 |
|  | Enalapril | 68(27.9) | 33(29.2) | 101(28.3) |  |
|  | Lisnopril | 51(20.9) | 14(12.4) | 65(18.2) |  |
| Beta-blockers |  | 26(10.7) | 18(15.9) | 44(12.3) | 0.159 |
|  | Metoprolol | 21(8.6) | 15(13.3) | 36(10.1) |  |
|  | Atenolol | 3(1.2) | 2(1.8) | 5(1.4) |  |
|  | Propranolol | 2(0.8) | 1(0.9) | 3(0.8) |  |
| Calcium channel blockers | | 43(147.6) | 28(24.8) | 71(19.9) | 0.115 |
|  | Nifedipin | 35(14.3) | 9(8.0) | 44(12.3) |  |
|  | Amlodipine | 8(3.3) | 19(16.8) | 27(7.6) |  |
| Diuretics |  | 45(18.5) | 18(15.9) | 63(17.6) | 0.567 |
|  | Hydrochlorothiazide | 29(11.9) | 12(10.6) | 41(11.4) |  |
|  | Furosemide | 16(6.6) | 6(5.3) | 22(6.2) |  |
| Lipid lowering agents (Statins) | | 141(58.3) | 55(48.7) | 196(46.0) | 0.090 |
|  | Simvastatin | 63(25.8) | 32(28.3) | 105(29.4) |  |
|  | Atorvastatin | 31(12.7) | 11(9.7) | 20(5.6) |  |
|  | Lovastatin | 26(10.7) | 6(5.3) | 20(5.6) |  |
|  | Rosuvastatin | 21(8.6) | 6(5.3) | 15(4.2) |  |
| Antiplatelets |  | 124(51.0) | 42(37.2) | 166(46.5) | 0.015 |
|  | Aspirin | 122(50.0) | 41(36.3) | 163(45.7) |  |
|  | Clopidegrole | 2(0.8) | 1(0.9) | 3(0.8) |  |
| **Others*** |  | 26(31.0) | 8(20.0) | 34(27.4) | 0.089 |
| OGLD: oral glucose-lowering drugs, * Antiasthmatics, proton pump inhibitors, and antineuropathics, drugs for thyroid | | | | | |
